# An Effect Analysis of Comprehensive Treatment of Groundwater Over-Exploitation in Cheng’an County, Hebei Province, China

**DOI:** 10.3390/ijerph14010041

**Published:** 2017-01-04

**Authors:** Weiwei Shao, Jinjun Zhou, Jiahong Liu, Haixing Zhang, Jianhua Wang, Chenyao Xiang, Guiyu Yang, Yun Tang

**Affiliations:** 1 State Key Laboratory of Simulation and Regulation of Water Cycle in River Basin, China Institute of Water Resources and Hydropower Research, Beijing 100038, China; shaoww@iwhr.com (W.S.); cyzjjfq@126.com (J.Z.); wjh@iwhr.com (J.W.); xiangchenyao@foxmail.com (C.X.); guiyuy@iwhr.com (G.Y.); tangyun@iwhr.com (Y.T.); 2 Dalian Waterwood Engineering Co. Ltd., Dalian 116021, China; zhx112700@163.com

**Keywords:** Cheng’an County, groundwater over-exploitation, comprehensive treatment, effect evaluation

## Abstract

The comprehensive treatment project of groundwater over-exploitation in Hebei Province has been implemented for more than a year, and the effect of exploitation restriction is in urgent need of evaluation. This paper deals with Cheng’an County of Hebei Province as the research subject. Based on collected hydro-meteorological, socioeconomic, groundwater, and other related data, together with typical regional experimental research, this study generates the effective precipitation–groundwater exploitation (P-W) curve and accompanying research methods, and calculates the quantity of groundwater exploitation restriction. It analyzes the target completion status of groundwater exploitation restriction through water conservancy measures and agricultural practices of the groundwater over-exploitation comprehensive treatment project that was implemented in Cheng’an County in 2014. The paper evaluates the treatment effect of groundwater over-exploitation, as well as provides technical support for the effect evaluation of groundwater exploitation restriction of agricultural irrigation in Cheng’an County and relevant areas.

## 1. Introduction

In the past 50 years, the changing spatial and temporal distribution of precipitation, river runoff volume reduction, and increasingly serious water shortages in northern China have put forward new requirements for agricultural irrigation water sources and irrigation methods [1,2]. The North China Plain, one of the most important agricultural regions in northern China, is currently faced with serious water resource shortages [3]. Its agricultural water relies on groundwater to a great extent. As the imbalance of water supply and demand worsens, the groundwater over-exploitation problem has become increasingly prominent. As an important part of the social water cycle, groundwater exploitation not only affects the hydrological cycle, but also has an impact on the hydrogeological conditions of the region. Groundwater exploitation quantities in an area should be subject to allowable exploitation limits. When the exploitation quantity exceeds the allowable amount, groundwater over-exploitation occurs, leading to a variety of geological, environmental, and social issues, including the drainage of the aquifer, land subsidence, karst collapse, deterioration of water quality, salt water intrusion (in coastal areas), etc. [4]. The groundwater over-exploitation area of the Hebei plain region accounts for 91% of the province’s plain areas. The groundwater over-exploitation is severe and has resulted in the partial drainage of the first aquifer in the Piedmont Plain area of Hebei, the groundwater depression cone, and a large area of land subsidence in the plain areas. In addition, the depression of the seawater brackish surface has appeared in parts of Cangzhou City and Hengshui City, meanwhile plain rivers are drastically blanking and drying up. There are additional significant deterioration issues in terms of the ecological environment.

Domestic and foreign scholars have completed a great deal of research on the impact of groundwater over-exploitation on the ecological environment and environmental geology. Pang et al. [5] utilized a two-dimensional finite element model to study the land subsidence changes caused by groundwater over-exploitation in the North China Plain. Hu et al. [6] used a comparison of groundwater exploitation quantity and exploitable quantity to quantitatively describe land subsidence, ground fissures, and other environmental issues caused by groundwater over-exploitation. Boling [7], Holzer [8], Wolff [9], Rothenburg [10], Wang et al. [11], and Wu et al. [12] studied the relationship between groundwater over-exploitation and ground fissures. Shi et al. [13] calculated and analyzed the degree of the over-exploitation of deep groundwater in the North China Plain by comparing the available quantity and exploitation quantity. Lee et al. [14] studied seawater intrusion problems in the Korean coastal plains caused by groundwater over-exploitation based on the data of 45 monitoring wells. He et al. [15] studied the impact of groundwater over-exploitation on the karst collapse. Scholars have also carried out related research studies on the groundwater dynamic change mechanism and its impact mechanism on crops. Jia et al. [16] estimated the over-exploitation quantity of shallow groundwater in the Haihe River Basin since 1959 by using ARCGIS technology based on the principle of the drainage method. Facchi et al. [17] simulated and studied the characteristics of the temporal and spatial distribution of crop water requirements and dynamic changes of groundwater against irrigation water in Italy’s alluvial plains shifting from the surface to the underground. Zhou et al. [18] studied the impact of groundwater over-exploitation on agricultural irrigation and proposed countermeasures. With increasing awareness of the severity of groundwater over-exploitation, scholars have also proposed a series of exploitation restrictive measures. Karatzas et al. [19] proposed that some wells must be closed to control seawater intrusion against the problems of seawater intrusion facing the Greek island of Crete caused by groundwater over-exploitation. Li [20] and Liu et al. [21] put forward restrictive measures of groundwater exploitation by shutting down and closing irrational groundwater exploitation engineering, constructing surface water conservancy projects, and introducing surface alternative water sources. Qi [22] engaged in multiple measures of water distribution by way of the South-to-North Water Diversion project, the closure of groundwater wells, sewage treatment, and recycling, as well as water-saving projects to carry out groundwater exploitation restrictions for Langfang City. However, regarding the effect of evaluations of groundwater over-exploitation treatment, there are, currently, relatively few research results and the research system is in need of further development. Zhang et al. [23] took a GIS-based evaluation of the potential development capabilities and comprehensive treatment effect of shallow groundwater in the Yellow River Delta. Tian et al. [24] evaluated the effect of groundwater exploitation restrictions in Nantong City of Jiangsu Province through considering the change of groundwater levels and the recovery of land subsidence. Xu et al. [25] evaluated the reasonable exploitation quantity of groundwater by regression analysis based on the data of land subsidence and groundwater extraction, and also analyzed the treatment effect of groundwater over-exploitation restrictions. You et al. [26] analyzed the impact of China’s South-to-North Water Diversion project for groundwater recharge in North China. Kumar et al. [27] proposed to evaluate the groundwater exploitation quantity by the type and number of wells.

In early 2014, China issued the No.1 document, which demanded that researchers “carry out the experimental work of the comprehensive treatment of groundwater over-exploitation in Hebei Province, North China in advance”. Hebei became a national pilot province for the comprehensive treatment of groundwater over-exploitation. Since 2014, Hebei Province has carried out the comprehensive treatment of groundwater over-exploitation in its plain areas, and further focused on the treatment of groundwater over-exploitation in four prefecture-level cities (i.e., Hengshui, Cangzhou, Handan, and Xingtai) covering 49 counties. Regarding the comprehensive treatment of groundwater over-exploitation in Hebei Province, experts and scholars have also put forward a series of measures and proposals. Zhang et al. [28] proposed a treatment idea with the intention to “give priority to major tasks and identify key issues”. Liu [29] proposed the “5 + 1” comprehensive treatment mode for groundwater over-exploitation (i.e., one core objective system and five innovative mechanisms). Xie et al. [30] discussed the agricultural water-saving systems, water-saving management systems, and industrial and urban domestic water saving measures in Guantao County. To be specific, it includes water-saving irrigation projects, agronomic water-saving measures, water rights management system, water pricing, and water fee collection systems, etc. Zhang et al. [31] and Wang et al. [32] explored the comprehensive treatment measures of groundwater over-exploitation. In 2014, all the pilot counties for the comprehensive groundwater exploitation restriction in Hebei Province have developed implementation plans of water conservancy measures and agricultural measures for the comprehensive treatment of groundwater over-exploitation. At present, the comprehensive treatment of groundwater over-exploitation in Hebei Province has been fully implemented for one year. The evaluation of its effect on groundwater exploitation restrictions is a critical work for the comprehensive treatment of groundwater over-exploitation in Hebei Province. A scientific and rational evaluation on the effect of groundwater exploitation restrictions will provide scientific reference for the further implementation of comprehensive treatment for groundwater over-exploitation. This study quantitatively evaluated the effect of groundwater exploitation restrictions in Cheng’an County, Handan City, Hebei Province based on hydrological and meteorological data, agricultural electricity consumption, ground water levels, and other basic information; the considerations presented in this paper will provide technical support for the evaluation of the 2014 comprehensive treatment of groundwater in Cheng’an. This study will also serve as a reference for groundwater protection in Cheng’an and similar regions.

## 2. Study Area and Data

Cheng’an County of Hebei Province is located at latitude 36°18′–36°30′ N, longitude 114°29′–114°53′ E. It is found in southern Hebei Province, southeast of Handan City, and east of Taihang Mountain. The total area of the county is 481.5 km^2^, consisting of four towns and five townships. Cheng’an County focuses on agricultural production. As a major grain-producing county in Hebei Province, agriculture dominates the county’s economic development. In 2014, the county’s total population was 443,000, of which the agricultural population accounted for 65.6%. The arable land in the county is 38,426.6 ha, of which 34,913.3 ha is basic irrigated farmland area. The crop types include food crops, cash crops, and others. Among the food crops, the summer grain’s majority production is wheat, whereas in autumn it is corn. Of the cash crops, cotton is the majority production product, while other crops include vegetables, fruits, etc. Cheng’an County is located in the alluvial plain area of the ancient Yellow and Zhanghe River, but lacks surface water resources within the territory. There is no natural river and groundwater acts as a major long-term industrial, agricultural, and domestic water source. Cheng’an County has relatively simple topography, with relatively flat terrain. Its soil type is mainly brown earth. Since the 1960s, Cheng’an County has long been overexploiting groundwater to maintain economic and social development, which has triggered a series of environmental and geological problems, such as shallow and deep groundwater depression cones, land subsidence, etc. The predatory exploitation of groundwater has long been a serious threat to regional water resources and water environment security, which, as a result, forms a 17 km^2^ shallow groundwater funnel area around the county and an approximate area of 60 km^2^ of deep groundwater over-exploitation in the northeast county.

In 2014, after being identified as a pilot county for groundwater over-exploitation in Hebei Province, Cheng’an County centered itself around “broadening water resources and reducing expenditure”, and has implemented efficient water-saving irrigation projects in the well irrigation areas, as well as surface water irrigation projects regarding water conservancy projects. Regarding agricultural projects, the county has implemented the adjustment of cultivation structure modes, water saving and stable production supporting technologies (spring irrigation for winter wheat), conservation tillage, fertilizer integration technology (wheat, corns, vegetables), as well as other agricultural groundwater exploitation restriction projects. In addition, combined with agricultural water pricing reform, water pipe structural reform, and other mechanism system constructions, as well as industrial and urban domestic groundwater exploitation restrictions, the county has carried out a series of comprehensive treatments for groundwater over-exploitation. This paper mainly evaluates and analyzes the completion effect of the water conservancy projects and agricultural projects implemented by Cheng’an County for the exploitation restrictions of agricultural groundwater in 2014.

**Figure 1:**
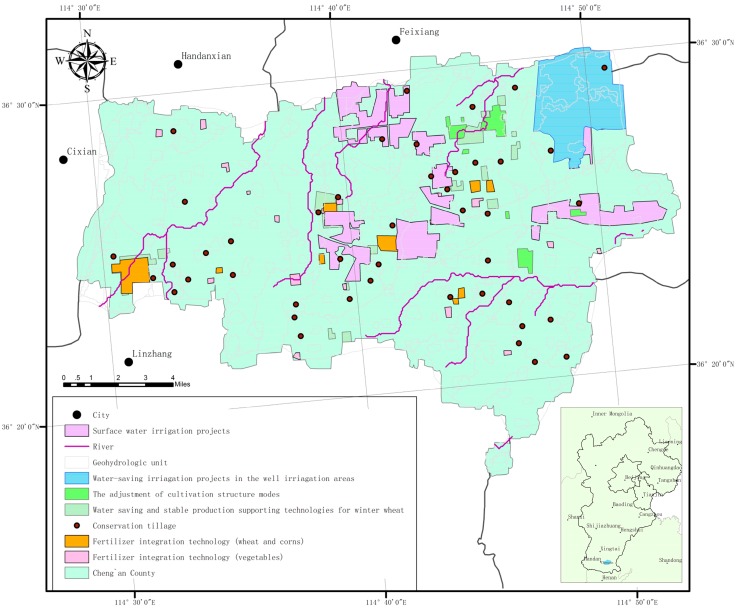
Distribution map of groundwater exploitation restriction projects in Cheng’an County.

The implementation of comprehensive treatment projects for groundwater over-exploitation in Cheng’an County in 2014 is as follows (Figure 1): The irrigation area of surface water replacing groundwater reached 3733.33 ha, including Daodongbu Township, Xinyi Township, etc., with a total of 42 villages. The irrigation area of efficient water-saving projects in the well irrigation areas reached 1686.67 ha, including Beixiangyi Township, with a total of 14 villages. The planting area of cultivation mode adjustment reached 400 ha, including Zhongganluo Village and Xinji Village in Zhanghedian Town where deep groundwater over-exploitation was severe, and also including Dadixi Village, Zhouhuadian Village, and 10 other villages in Daodongbu Township where shallow groundwater over-exploitation was especially serious. The spring irrigation water-saving area for winter wheat reached 706.66 ha, including Beixiangyi Township, Xinyi Township, and other three other townships, with a total of more than 20 villages. The wheat-corn fertilizer integration project covered an area of 266.67 ha, including nine villages in Changxiang and Baisiying Townships, as well as three other townships. The conservation tillage project covered an area of 3333.33 ha, including 58 family farms and grain planting cooperation organizations all around the county. The fertilizer integration project for drip irrigation under the vegetable membrane covered an area of 213.33 ha, including 10 towns, townships, and 19 business entities.

The study collected socio-economic development data, agricultural information (such as planting structure, etc.), water resource basic data, a long series of annual precipitation data, groundwater depth information, agricultural electricity consumption data, and other basic information of Cheng’an County, wherein the socio-economic related data came from the Statistical Yearbook of Cheng’an County; the agricultural planting structure data came from Cheng’an County Agricultural Bureau; 2008–2015 Cheng’an County Water Resources Annual Report and 2013 Cheng’an County Water Conservancy Census Results provided by Cheng’an County Water Conservancy Bureau; the daily precipitation data of the Cheng’an rainfall station were obtained from the Hydrological Bureau of Hebei Province, dated between 1956 and 2015; the groundwater depth information was obtained from the Hydrological Bureau of Handan City, dated between 2006 and 2015 (nine groundwater observation points, two deep groundwater observation points, with monthly observation data). In addition, the study also collected four well-log data (both shallow wells) within the groundwater exploitation restriction project areas, of which, the three wells are located in the Baisiying and Zhanghedian Townships as well as Shangcheng Town, with 2013–2015 monitoring data (monitoring twice a year, in May and September, respectively). Another well is located in Changxiangying Township, with 2013 to 2015 monthly monitoring data; the monthly agricultural irrigation electricity consumption data in each town and township in 2013 and 2015 were provided by the Electric Power Bureau of Cheng’an County; the electricity data of groundwater extraction in the experimental areas were provided by the person in charge of each of the village wells.

**Figure 2:**
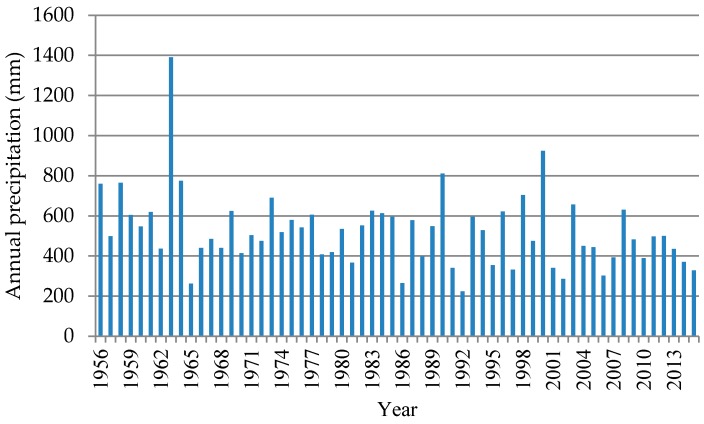
Variation in the annual amount of areal precipitation from 1956 to 2015 in Cheng’an County.

According to the statistical analysis, the total amount of water resources in Cheng’an County is 64.98 million m^3^, and the available water yield of water resources is 53.83 million m^3^. The inter-annual changes in precipitation are great in Cheng’an County, and the distribution within the year is uneven; approximate 73 percent of annual precipitation is concentrated in the flood season of June to September. From the change analysis of the average annual amount of areal precipitation from 1956 to 2015 in Cheng’an County rainfall station, it can be seen that rainfall showed a significant downward trend (Figure 2), with a multi-year average amount of precipitation of 521.8 mm. The rainfall reached a peak in 1963, reaching as high as 1390.6 mm; in 1992, the rainfall in Cheng’an County reached its lowest point in nearly 50 years, at only 224.2 mm.

## 3. Research Methods

The comprehensive treatment for groundwater over-exploitation in Cheng’an County was implemented in 2014. As such, 2013 was undertaken as the pre-treatment baseline year for the evaluation of groundwater exploitation restrictions, and 2015 was seen as the post-treatment baseline year. The actual exploitation quantity of agricultural groundwater is closely related to high and low precipitations within the year. In years with abundant rainfall, the crops use more precipitation and require less amounts of irrigation; otherwise, the crops use less precipitation, leading to an increased demand for irrigation. Therefore, the amount of groundwater exploitation should take into account precipitation factors. The agricultural water exploitation quantity in a region with certain crop structures and water consumption is generally measured by the groundwater exploitation quantity in the normal flow year, because it has the strongest representativeness and approximates the expected quantity of groundwater exploitation under changes in precipitations for many years. In this study, the actual groundwater exploitation quantities in the pre-treatment baseline year (2013) and in the post-treatment baseline year (2015) were converted to the exploitation quantities under the normal flow year by calculating the actual exploitation quantities of agricultural groundwater and the conversions of high and low exploitation quantities, in addition to the exploitation restriction quantity of groundwater which was obtained through comparison and analysis, thus evaluating the comprehensive treatment effect for groundwater over-exploitation.

### 3.1. Selection of High, Normal, and Low Flow Representative Years in the Evaluated Regions

In this study, the effective precipitation is regarded as actual rain-fed for agriculture. Effective precipitation is the amount of rainfall that infiltrates the soil and is stored in the water absorption layer of the main root of the crop [33]. It equals the amount of the precipitation that remains after surface runoff and deep percolation are deducted. It is affected by the depth of the water absorption layer of the root system, water holding capacity of the soil, antecedent soil water storage, rainfall intensity, rainfall amount, and other factors [34]. The article proposes that effective precipitation is the deduction of the loss of precipitation, and directly supplements precipitation for the growth of the crops. The effective precipitation was analyzed through areal precipitation from 1956 to 2015 in Cheng’an County. The effective precipitation was calculated according to the following formula: $P_{0}=\;\propto P$, wherein, *P*_0_ is the effective precipitation (mm); *P* is the precipitation event (mm); ∝ is the coefficient of precipitation infiltration, which is related to the precipitation event, precipitation intensity, duration, soil properties, ground cover, terrain, and other relevant factors. It is generally believed that when the precipitation event is less than 5 mm, ∝ is 0; when the precipitation event is 5–50 mm, ∝ is 0.8 to 1; when the precipitation event is greater than 50 mm, ∝ is 0.7 to 0.8 [35,36]. It is known through analysis that the multiyear average effective precipitation of Cheng’an County is 311 mm. The Pearson III type curve was adopted for the calculation of experimental frequency curve, and the fitting degree R^2^ was not less than 0.92. Based on effective precipitation quantity, the high, normal, and low representative years within these recent 10 years were 2009, 2010, and 2007, respectively, when effective precipitation quantities were 377.07 mm, 341.43 mm, and 303.20 mm respectively.

### 3.2. Field Experiments of Exploitation Quantity per Kilowatt Hour for a Single Well in a Typical Zone

Taking into account the differences that may exist between the groundwater exploitation restriction areas and the non-project areas, as well as the regional variability of exploitation quantity per kilowatt hour in a single well that may exist between different towns and townships, the experiment selected multiple typical zones for observation. They covered the hydraulic exploitation restriction project area and the agricultural exploitation restriction project area in Cheng’an County. The main planting structure of a typical zone is a wheat maize rotation. The irrigation method is mostly pipe irrigation and water resources are taken from shallow groundwater. In the typical zones of non-project areas and different types of project areas, the pumping well tests were carried out separately. Eight wells were selected in the project areas and two wells in the non-project areas were also selected. The irrigation extraction volume and electricity consumption within half an hour were recorded and the record interval was 6 min. Accordingly, groundwater exploitation quantity per kilowatt hour in a single well was calculated. The measurement results showed that the average exploitation quantity per kilowatt hour in a single well in the non-project areas was 2.89 m^3^; the exploitation quantity per kilowatt hour in a single well in the Water Conservation Projects of well irrigation districts, fertilizer integration projects, spring irrigation for winter wheat and water conservation projects were 2.52 m^3^, 2.37 m^3^, and 1.33 m^3^ respectively. Combined with the irrigation electricity consumption data of wells in the high, normal, and low representative years, as well as the irrigation area of the wells, the water consumption per ha of main crops in the high, normal, and low flow representative years in typical zones were obtained through analysis.

### 3.3. P-W Curve Plotting

**Figure 3:**
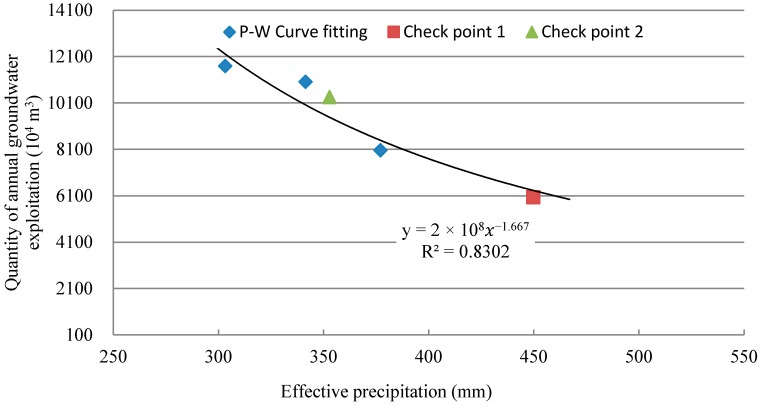
The precipitation–groundwater exploitation (P-W) curve analysis in Cheng’an County.

According to the agricultural electricity consumption data and pumping quota outlining every single kWh from 2007 to 2015 in Cheng’an County, combined with the agricultural planting structures, the quantity of the groundwater exploitation was analyzed and calculated in different years. According to the effective precipitation in high, normal, and low flow representative years (2009, 2010, and 2007, respectively), and using 2008 and 2012 as the check-point years, the precipitation–groundwater exploitation (P-W) curve of effective precipitation (P) and groundwater exploitation quantity (W) was plotted (Figure 3), the power equation of the P-W curve was fitted, and equation y = 2 × 10^8^x^−1.667^ was obtained, wherein, y is the quantity of annual groundwater exploitation, x is an annual effective precipitation, and the correlation coefficient R^2^ was 0.83.

### 3.4. Calculation of Exploitation Restriction Quantity

The amount of precipitation in a normal flow year refers to the amount of precipitation at 50% frequency time in an effective precipitation series from 1956 to 2013 in Cheng’an County. The quantity of groundwater exploitation corresponding to $P_{50}$ can be found in the plotted P-W curve, namely $W_{2013}^{Med}$ (ten thousand m^3^).

It is assumed that there are *N* well irrigation zones/well and canal irrigation zones in the post-treatment baseline year (2015) in the project area, and there are *S* planting types of crops in each irrigation zone using groundwater irrigation. If so, the actual quantity of groundwater exploitation in the post-treatment baseline year (2015) is:

(1) $$W_{2015}^{ACT}=\sum_{i=1}^{N}\sum_{j=1}^{S}\left(w_{i,j}^{2015}\cdot a_{i,j}^{2015}\right)$$

where $a_{i,j}^{2015}$ is the area (ha) of well irrigation of type *j* crop in irrigation zone *i* in the post-treatment baseline year (2015), and $w_{i,j}^{2015}$ is the exploitation (m^3^/ha) quantity per unit area of type *j* crop in irrigation zone *i* in the post-treatment year (2015).

The quantity of groundwater exploitation in the normal flow condition in the post-treatment baseline year (2015) was determined according to the quantity of groundwater exploitation in the normal flow condition in the pre-treatment baseline year (2013), the actual quantity of groundwater exploitation in the checking baseline year (2015), the amount of precipitation in the post-treatment baseline year (2015), and the P-W curve. The formula is:

(2) $$W_{2015}^{Med}=W_{2013}^{Med}\times \frac{W_{2015}^{ACT}}{W_{2013}^{PW,2015}}$$

where $W_{2015}^{Med}$ is the quantity (ten thousand m^3^) of groundwater exploitation in the normal flow condition in the post-treatment baseline year (2015); $W_{2013}^{\mathrm{PW},2015}$ is the quantity (ten thousand m^3^) of groundwater exploitation in the pre-treatment baseline year (2013), and the P-W curve corresponds to the amount of precipitation in the post-treatment baseline year (2015).

The quantity of the groundwater exploitation restriction in the post-treatment baseline year (2015) is based upon the difference of the quantity of the groundwater exploitation in the normal flow condition in the pre-treatment baseline year (2013). The quantity of the groundwater exploitation in the normal flow condition in the post-treatment baseline year (2015) is determined by:

(3) $$W_{2015}^{\mathrm{Res}}=W_{2013}^{Med}-W_{2015}^{Med}$$

where $W_{2015}^{\mathrm{Res}}$ is the quantity of groundwater exploitation restriction in the post-treatment baseline year (2015) (ten thousand m^3^). Other symbols mean the same as previously stated.

## 4. Results and Discussion

### 4.1. Analysis of the Actual Quantity of Groundwater Exploitation in the Pre-Treatment and Post-Treatment Baseline Years

Together with the statistical data of Cheng’an County Statistical Yearbook and Cheng’an County Water Resources Annual Report in 2013, as well as the planting structure of Cheng’an County in 2013 and the data of exploitation quantity per kilowatt-hour for a single well in non-project areas in the typical zone field experiments, the actual quantity of groundwater exploitation in Cheng’an County in the pre-treatment baseline year in 2013 was obtained. The 2013 actual exploitation quantity of agricultural groundwater in the area corresponding to the implementation area of the exploitation restriction project (project area) in 2014 in Cheng’an County was 26.8191 million m^3^.

The Cheng’an County Statistical Yearbook and Water Resources Annual Report of 2015 have not yet been published. According to the data of the agricultural planting structure and planting area of Cheng’an County in 2015, as well as the electricity consumption of irrigation in Cheng’an County in 2015, the quantity of the groundwater exploitation per unit area of Cheng’an County in 2015 was calculated (Table 1). The actual quantity of groundwater exploitation in the project area and non-project area in the 2015 planting structure in Cheng’an County were additionally obtained through the statistics. Accordingly, the total quantity of agricultural groundwater exploitation in the project area in Cheng’an County in 2015 was 27.6226 million m^3^.

### 4.2. The Effect Evaluation of the Quantity of Groundwater Exploitation Restrictions

**Figure 4:**
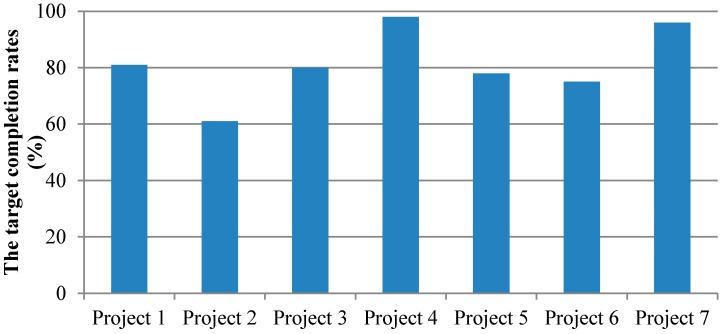
The target completion rates of the groundwater exploitation restriction projects. Project 1 represents highly efficient water saving projects in the well irrigation area; Project 2 represents surface water substitutes for groundwater engineering; Project 3 represents adjustment of planting modes; Project 4 represents conservation tillage; Project 5 represents spring irrigation for winter wheat (water saving and stable production); Project 6 represents water and fertilizer integration (vegetables); and Project 7 represents water and fertilizer integration (wheat and corn).

The quantities of groundwater exploitation under normal conditions in the pre-treatment baseline year (2013) and the post-treatment baseline year (2015) in Cheng’an County were 29.9207 million m^3^ and 18.3034 million m^3^, respectively, after calculation. The quantity of groundwater exploitation in the project area was restricted by 11.6173 million m^3^ compared to that in the pre-treatment year. The quantities of exploitation restrictions in the normal flow year in various projects obtained through analysis were compared with the corresponding target quantities of exploitation restrictions in the implementation plans of the comprehensive treatment of groundwater over-exploitation in various projects throughout 2014. Furthermore, the completion rates of the comprehensive treatment of groundwater over-exploitation in various projects in Cheng’an County can be obtained (Figure 4). In Figure 4, project 1 represents highly efficient water saving projects in the well irrigation area, project 2 represents surface water substitutes for groundwater engineering, project 3 represents adjustment of planting modes, project 4 represents conservation tillage, project 5 represents spring irrigation for winter wheat (water saving and stable production), project 6 represents water and fertilizer integration (vegetables), and project 7 represents water and fertilizer integration (wheat and corn). From the Figure, it can be seen that the completion rates of all exploitation restriction projects in Cheng’an County in 2015 were satisfactory. The completion rate of agricultural measures is slightly higher than that of water conservancy projects. In terms of the surface water replacement of groundwater projects, as the current supporting engineering facilities of water diversion project have not yet been completed, the completion rate is relatively low.

### 4.3. Analysis of the Recovery Effect of Groundwater Depth before and after Treatment

#### 4.3.1. Variation of Groundwater Depth in the Project Areas

**Figure 5:**
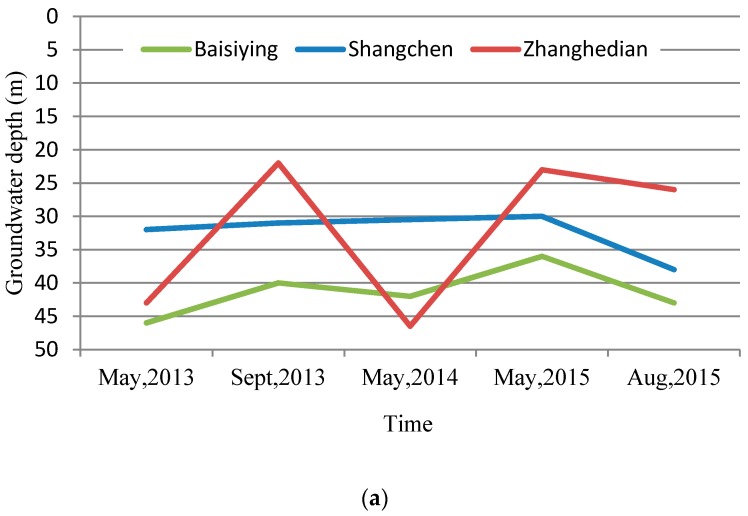

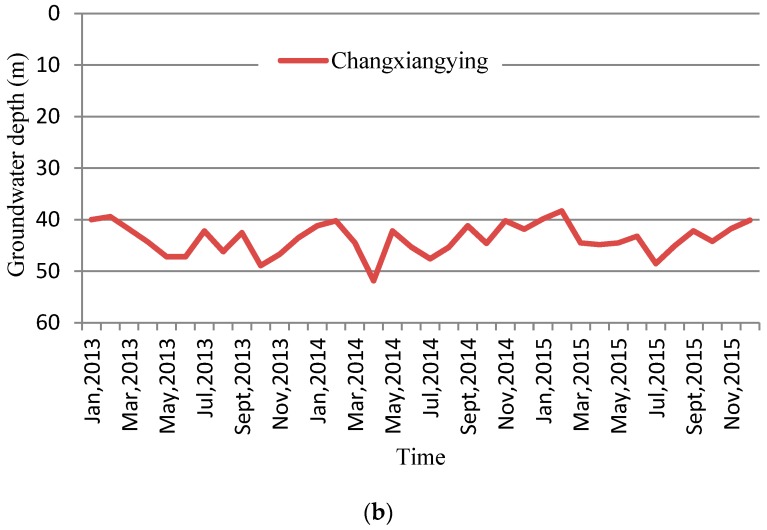
Comparison of the groundwater depth before and after treatment in the project areas. (**a**) The well-logs in Baisiying and Zhanghedian Townships as well as Shangcheng Town; (**b**) The well-log in Changxiangying Township.

Based on the well-log data in the exploitation restriction project areas, combined with the calculated data of the groundwater exploitation quantity in the project areas of this evaluation report, the recovery effect of the groundwater depth in the project area before and after treatment was analyzed. The well-log in Baisiying Township is located in the wheat and corn water and fertilizer integration project area. The well-log in Zhanghedian Township is located in the surface water replacement project area. The well-log in Shangcheng Town east is located in the vegetable film drip irrigation water and fertilizer integrated project area. Lastly, the well-log in Changxiangying Township is located in the winter wheat water-saving and stable production project area. The analysis of the variation of groundwater depths in the project areas shows that the groundwater depths of three well-logs in Baisiying and Zhanghedian Townships as well as Shangcheng Town in the project areas in May 2015 were higher than that of May 2013, with measured depths that were 10 m, 20 m, and 2 m higher respectively. Additionally, the groundwater level was restored to some extent (Figure 5). The well-log analysis of Changxiangying Township shows that the groundwater depth of the well-log after treatment by the groundwater exploitation restriction project was restored, to some extent, from an average depth of 44.2 m in 2013 to 43.1 m in 2015. From the view of the groundwater exploitation quantities before and after treatment in the project areas calculated in this study, the change of groundwater depths was consistent with the research results, and the quantities of groundwater exploitation were relatively reduced in the project areas.

#### 4.3.2. Variation of Groundwater Depth across the County

**Figure 6:**
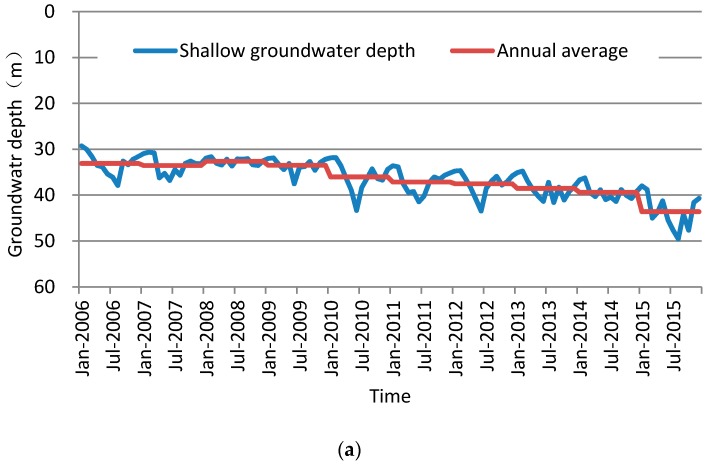

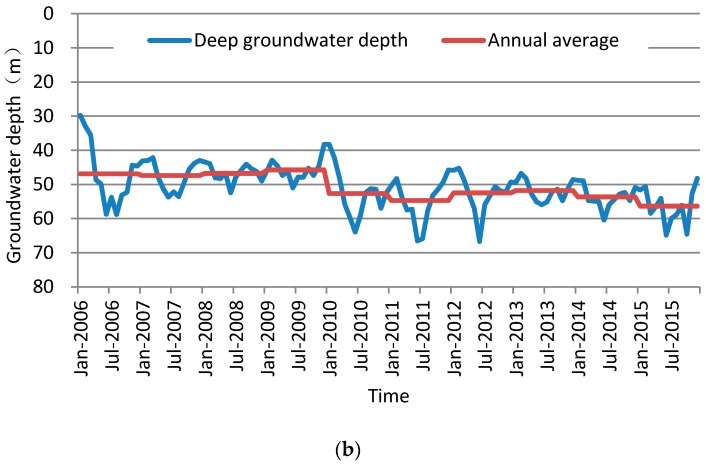
Variation of groundwater depth from 2006 to 2015 in Cheng’an County: (**a**) Shallow; (**b**) Deep.

Based on the average areal groundwater depth of Cheng’an County from 2006 to 2015, the shallow and deep groundwater depth map of Cheng’an County was plotted. It can be seen that after the implementation of the comprehensive treatment of groundwater exploitation restrictions, the groundwater depth in the project area maintained an upward trend, however, the county’s shallow and deep groundwater levels were still falling, as shown in Figure 6. This shows that although the groundwater level had been restored compared to how it was prior to the exploitation restriction measures that were taken, the restoration effect of the groundwater level is revealed due to the relatively small proportion of the groundwater exploitation restriction project areas. The groundwater depth is still falling across the county and the ecological restoration of groundwater in Cheng’an County remains a focus for further initiatives.

### 4.4. Shortcomings and Limitations

The study made a quantitative evaluation on the effect of the groundwater exploitation restriction for the comprehensive treatment of the groundwater over-exploitation of Cheng’an County, Hebei Province based on the hydro-meteorological data, agricultural electricity consumption, groundwater levels, etc. However, this study has its own shortcomings and limitations. First, due to time and energy constraints, this study failed to carry out the pumping experiments to test water yields per kilowatt hour for all of the wells in the project areas. Instead, the sampling experiment was carried out in a few typical areas. As a result, inconsistency of the water yields for some wells may be ignored. Second, considering the comprehensive treatment project on groundwater over-exploitation has been running for just one year when this research began, the groundwater level has not yet obviously risen, while the water table in the non-project area has not displayed a rise due to the small percentage of project area against the overall area. Nevertheless, there will be subsequent research studies concerning the groundwater over-exploitation status. Third, the study area in this paper is limited for the reasons surrounding data collection and difficulties associated with the arrangements of experiments, but in the future, the authors will try to conduct a study on a wider scale, such as the North China Plain, using the same evaluation method. 

## 5. Conclusions

This study involved collection of 58 years (1956–2013) of rainfall data from Cheng’an County for hydrological frequency analysis, and agricultural irrigation power data (2007–2015). The P-W curve was established from the data of the three years closest to high, normal, and low flow representative years between 2007 and 2013. Then the relationship between agricultural electricity consumption and groundwater exploitation quantity was determined through field experiments, including the various pressure water extraction projects and non-project conditions. Based on the P-W curve, the quantities of the groundwater exploitation of the pre-treatment baseline year (2013) and the post-treatment baseline year (2015) were converted to the quantities of groundwater exploitation in the normal flow year. Finally, their differences were compared to determine the quantities of exploitation restrictions of the comprehensive treatment project for groundwater over-exploitation in Cheng’an County.

The results show that the quantity of exploited groundwater in the project area was restricted by 11.6173 million m^3^ between 2013 and 2015. Among them, projects related to conservation tillage and water and fertilizer integration have had a higher completion rate (>90%), while the surface water replacement of groundwater projects have had a lower rate, which is just 60%. On the other hand, the project area groundwater level rise data also shows the comprehensive treatment of groundwater over-exploitation to obtain effects. This shows that treatment of groundwater over-exploitation in Cheng’an County has been effective, but some of the projects such as the surface water replacement project still have the problem of low efficiency.

This study proposes a method for calculating the quantity of groundwater exploitation restrictions by converting agriculture irrigation electricity consumption into water volumes. This method took into account the influences of natural rainfall on agricultural irrigation water consumption, realized the quantitative evaluation of groundwater exploitation restrictions, and accordingly provided some technical support for the comprehensive treatment of the groundwater over-exploitation in Hebei Province and similar regions.

## Figures and Tables

**Table 1:** Actual quantity of agricultural groundwater exploitation in Cheng’an County in 2015 (in the project areas).

Type	Implemented Project	Planted Crops	Planting Area (ha)	Water Consumption per ha (m^3^/ha)	Exploitation Quantity of Agricultural Groundwater (Ten Thousand m^3^)
1	Surface water substitute for groundwater engineering	Wheat, corn	3733.33	1605 (groundwater)	599.57
2	Highly efficient water saving project in the well irrigation area	Wheat, corn	1686.67	3165	534.11
3	Adjustment of planting modes	Cotton, and others	400	2535	101.79
4	Spring irrigation for winter wheat, water saving, and stable production	Wheat	706.67	2715	191.61
5	Conservation tillage	Wheat	3333.33	3720	1244.58
6	Water and fertilizer integration	Wheat, corn	266.66	1560	41.97
		Vegetables	213.33	2265	48.63
Total			10,340		
